# Expression génitale isolée du syndrome de Stevens-Johnson

**DOI:** 10.11604/pamj.2023.44.126.38614

**Published:** 2023-03-14

**Authors:** Idriss Ziani, Ahmed Ibrahimi

**Affiliations:** 1 Service de Chirurgie Urologique “A”, Centre Hospitalier Universitaire Ibn Sina, Faculté de Médecine, Université Mohammed V de Rabat, Rabat, Maroc

**Keywords:** Syndrome de Stevens-Johnson, pronostic vital, atteinte génitale, Stevens-Johnson syndrome, life-threatening condition, genital involvement

## Abstract

Stevens-Johnson syndrome (SJS) is a dermatological and systemic disease. It is a potentially life-threatening condition, almost always triggered by medications, and characterized by extensive skin destruction. It is sometimes associated with the involvement of other epithelia. Its immunological mechanisms are only partially elucidated. Current hypothesis is: delayed-type drug hypersensitivity involving drug-specific cytotoxic T lymphocytes in the blood and skin of patients. Clinically, symptoms occur 1 to 3 weeks after the incriminated drug is started. The patient develops prodromal symptoms including a feeling of discomfort, fever, headache, cough, and keratoconjunctivitis. Macules, often showing a “cockade” pattern, suddenly appear. These lesions appear simultaneously elsewhere on the body, coalesce into large flaccid bullae and slough over a period of 1 to 3 days. Diagnosis is often based on lesions occurrence and rapid progression of symptoms. Histological examination of sloughed skin shows typical necrotic epithelium. Treatment is based on supportive care, cyclosporin, plasmapheresis or IV immunoglobulins, early corticotherapy and TNF (tumor necrosis factor)-alpha inhibitors. Mortality rate is high. The particularity of this case is the isolated genital involvement, 6 days after the intake of a non-steroidal anti-inflammatory drug. Despite prompt management in the Intensive Care Unit, the patient had multi-visceral failure. He died due to refractory shock.

## Image en médecine

Le syndrome de Stevens-Johnson (SSJ) est une affection dermatologique et systémique qui peut engager le pronostic vital, il est presque toujours d´origine médicamenteuse, caractérisé par une destruction étendue de l´épiderme, associée parfois à l´atteinte d´autres épithéliums. Les mécanismes immunologiques ne sont que partiellement élucidés. L´hypothèse actuelle est une hypersensibilité retardée avec présence de lymphocytes T cytotoxiques, spécifiques du médicament inducteur, dans le sang et la peau des patients. Sur le plan clinique, les symptômes apparaissent 1 à 3 semaines après le début de la prise du médicament incriminé, le patient développe un prodrome associant sensation de malaise, fièvre, céphalées, toux, et kératoconjonctivite. Des macules, souvent sous forme de “cocarde”, apparaissent alors brutalement, ces lésions apparaissent simultanément ailleurs sur le corps, confluent en de grandes bulles flaccides et se décollent sur une période de 1 à 3 jours. Le diagnostic est souvent établi grâce à l'apparence des lésions et à la progression rapide des symptômes. L'examen histologique de la peau décollée montre un épithélium nécrotique caractéristique. Le traitement repose sur les soins de support, la cyclosporine, la plasmaphérèse ou les immunoglobulines IV, la corticothérapie précoce et les inhibiteurs du *TNF* (*Tumor Necrosis Factor*)-alpha ont été utilisés. Le taux de mortalité reste élevé. La particularité de notre observation est l´atteinte génitale isolée, 6 jours suite à la prise d´un anti-inflammatoire non stéroïdien, malgré la prise en charge rapide en milieu de réanimation le patient a présenté rapidement une défaillance multi viscérale avec décès par un état de choc réfractaire.

**Figure 1:**
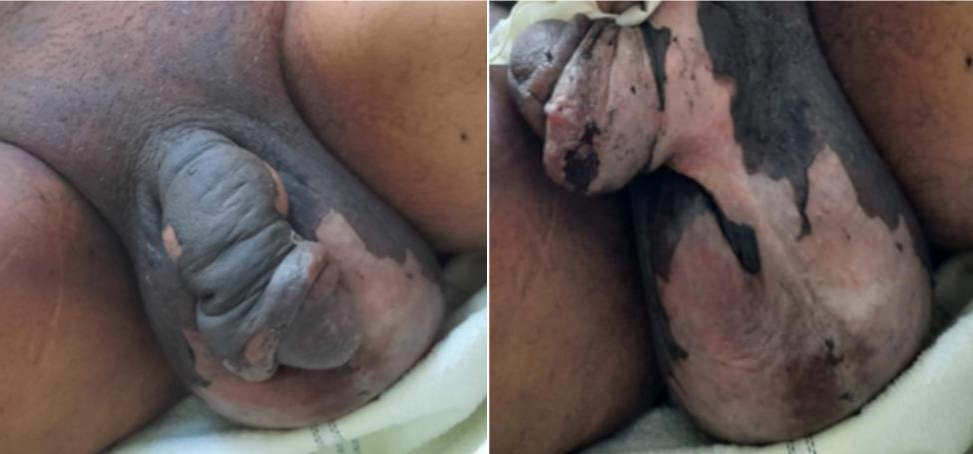
lésions cutanées polymorphes appelées lésions en cocarde atypiques (macule érythémateuse qui se décolle)

